# Borderline phyllodes tumour of the breast with eosinophilic inclusion bodies: Case report and molecular sequencing

**DOI:** 10.1016/j.ijscr.2023.108776

**Published:** 2023-09-07

**Authors:** Ngoc My Lam, Jean-Christophe Noël, Nicky d'Haene, Isabelle Salmon, Bruno Vandermeersch, Xavier Catteau

**Affiliations:** a Department of Pathology, CurePath (Chirec Institute-Brussels, CHU Tivoli-La Louviere), Rue de Borfilet 12A, 6040 Charleroi, Belgium; b Department of Pathology, Brussels University Hospital (HUB), Rue Meylemeersch 90, 1070 Anderlecht, Belgium; c Department of Gynecology, Ste-Anne St-Rémy Clinic, Bd Jules Graindor 66, 1070 Anderlecht, Belgium

**Keywords:** Phyllodes tumour, Borderline, Breast, Eosinophilic inclusion bodies, Molecular, Infantile digital fibromatosis

## Abstract

**Introduction and importance:**

The presence of eosinophilic inclusion bodies in the breast is very rare and fewer than 20 cases were described in the literature. Herein we report the first case of borderline phyllodes tumour associated with this kind of cells. To the best of our knowledge, this is also the first time that a molecular sequencing is made targeting the stroma cells with inclusion bodies.

**Case presentation:**

A 33-yr-old woman presented a large mass in the right breast. Imaging techniques by mammogram and ultrasonographic examination were performed. After multidisciplinary approach, a breast conserving surgery has been decided. Microscopic analysis, immunohistochemical stains and molecular tests were performed on the lesion. The proposed diagnosis is borderline phyllodes tumour with eosinophilic inclusion bodies.

**Clinical discussion:**

Inclusion bodies are typically found in the infantile digital fibromatosis. Finding them in extradigital fibromatosis is rare. Their signification is still unclear. Some studies suggest a disturbance in the metabolism of proliferating myofibroblasts.

**Conclusion:**

The presence of inclusion bodies in breast tumour do not seem to have a prognosis impact. It might be interesting to perform others molecular tests on lesions with eosinophilic inclusion bodies to discover potential mutations.

## Highlights

• Presence of eosinophilic inclusion bodies in the breast is very rare.
• First case of borderline phyllodes tumour associated with this kind of cells.
• First molecular sequencing targeting the stroma cells with inclusion bodies.

## 1 Introduction

Eosinophilic intracytoplasmic inclusion is a pathognomonic feature of infantile digital fibromatosis (IDF) [1]. It is a rare, superficial fibromatosis characterized by little, firm, painless, pink-red nodules on the fingers and toes of children. The pathogenesis of Idiopathic Digital Fibromatosis (IDF) is still unclear and many studies have not found an association with a genetic syndrome or systemic disease. Eosinophilic inclusion bodies in extradigital fibromatosis are uncommon. Some cases have reported intracytoplasmic inclusion bodies in cervical polyps [2], in leiomyosarcoma [3] or in phyllodes tumour of the breast [4]. To the best of our knowledge, we report for the first time a phyllodes tumour with borderline features associated with eosinophilic intracytoplasmic inclusions. This is also the first that a molecular sequencing is made in this context.

## 2 Case presentation

**Fig. 1:**
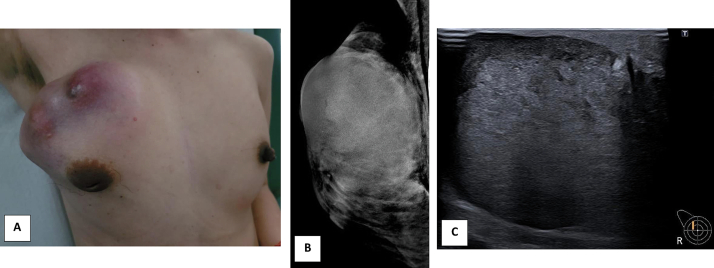
Clinical and imaging techniques. A. Mass with a bluish spot on the skin in the upper quadrant of the right breast. B. Mammogram C. Ultrasound of the lesion in the right breast.

Here we report a case of a 33-yr-old woman who presented a large mass in the right breast. Her surgical history includes a breast conserving surgery (BCS) in Cambodia in 2019 for a benign lesion without more precise details. She is not using contraception. In her family history, her mother's cousin had breast cancer at the age of 50. The breast examination revealed a large mass with a bluish spot on the skin in the upper external quadrant of the right breast (Fig. 1).

There was an enlargement of the areola and a retraction of the nipple. The examination of the left breast was unremarkable. Bilateral mammogram was performed and showed dense breasts. In the right breast, we found a mass measuring 8 cm and occupying the entire upper quadrant. The lesion was relatively well delimited. Rare microcalcifications were found but were not suspicious. In the left breast, there were scattered microcalcifications but no suspicious focus. Ultrasound of the right breast showed a vascularised mass measuring 15 × 4.7 cm (Fig. 1).

Microbiopsy was performed. Histologically, biopsy cores showed a biphasic tumour with a fibroepithelial proliferation and small glandular structures. The fibrous compartment was fibrous, focally myxoid and contained a few irregularly arranged fibroblasts. The proliferation index evaluated by Ki67 (MIB-1, Dako) was estimated at 10 %. The lesion was classified as B3 in the Nottingham system [5].

After multidisciplinary approach, a BCS has been decided. The patient had no post-operative complications.

**Fig. 2:**
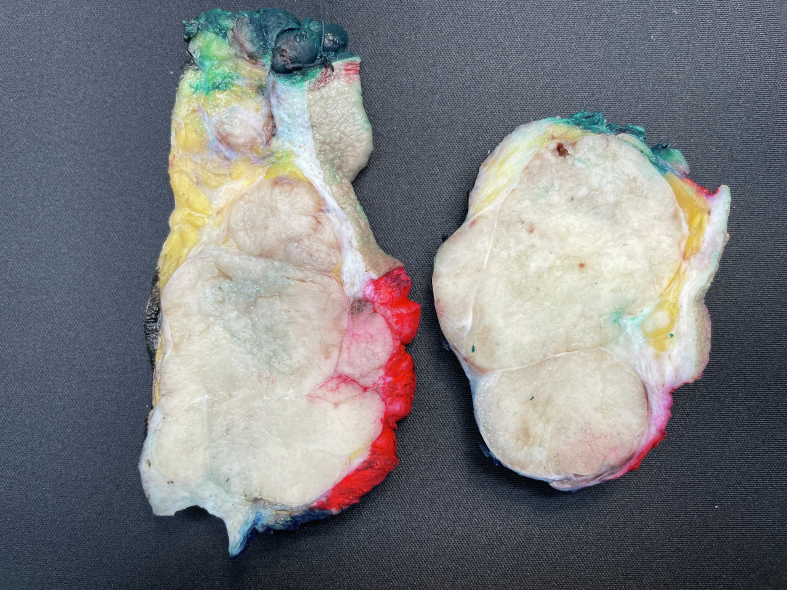
The lesion measuring 11 × 10 × 7 cm was mostly encapsulated and appeared multinodular, gray-white, homogenous at the cut surface.

Macroscopically, the resected lesion measured 11 × 10 × 7 cm, was relatively well delimited and appeared as a multinodular, gray-white, homogeneous mass on the cut surface (Fig. 2).

**Fig. 3:**
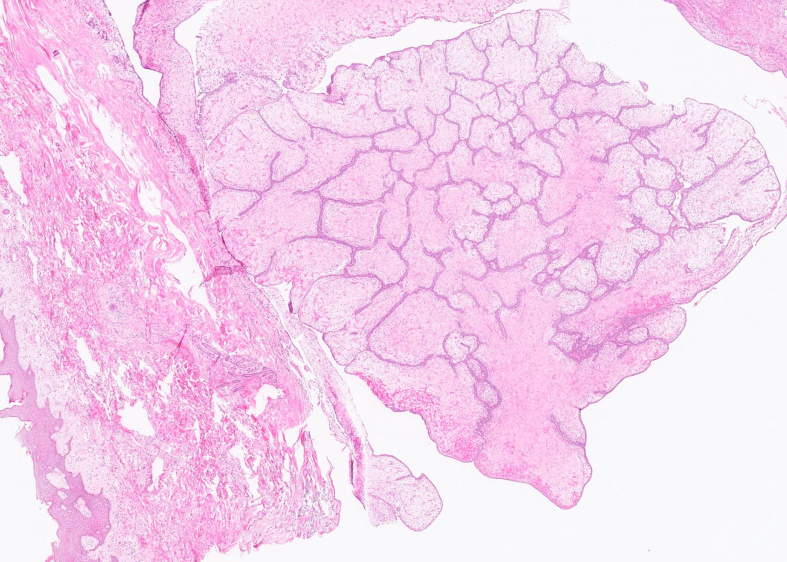
Microscopic examination. Fibroepithelial proliferation (HE, ×2).

**Fig. 4:**
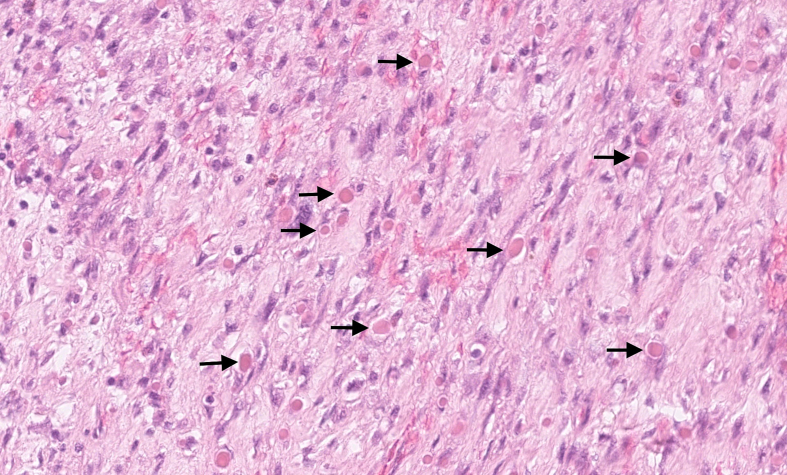
Microscopic examination. Proliferating stromal cells containing eosinophilic inclusion bodies (arrows) (HE ×40).

**Fig. 5:**
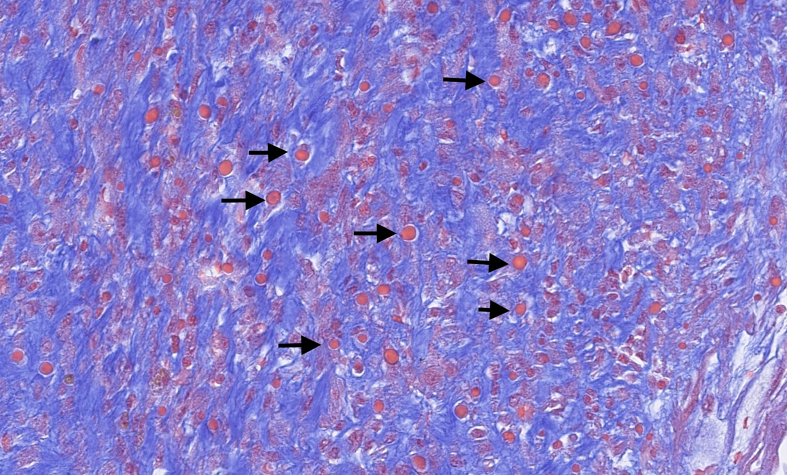
Inclusion bodies stained red by Masson's trichrome (arrows) (HE ×40). (For interpretation of the references to colour in this figure legend, the reader is referred to the web version of this article.)

**Fig. 6:**
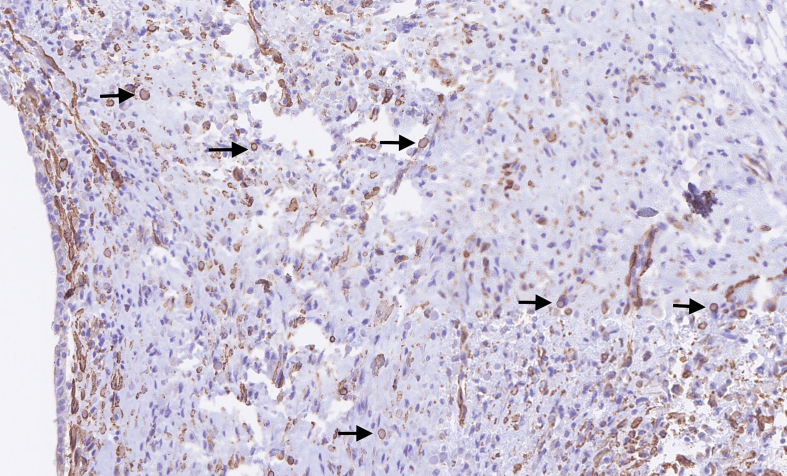
Immunohistochemical of the lesion and the inclusion bodies (arrows). Positivity for Smooth Muscle Actine (SMA) (×20).

**Fig. 7:**
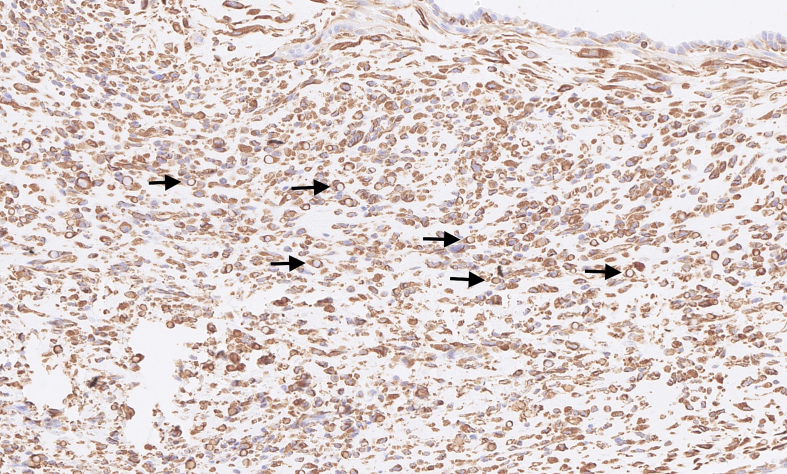
Immunohistochemical of the lesion and the inclusion bodies (arrows). Positivity for vimentin (×20).

**Fig. 8:**
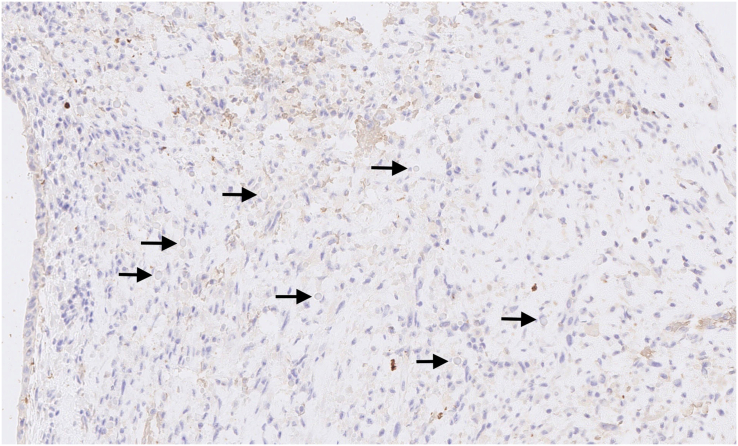
Immunohistochemical of the lesion and the inclusion bodies (arrows). Negativity for desmin (×20).

**Fig. 9:**
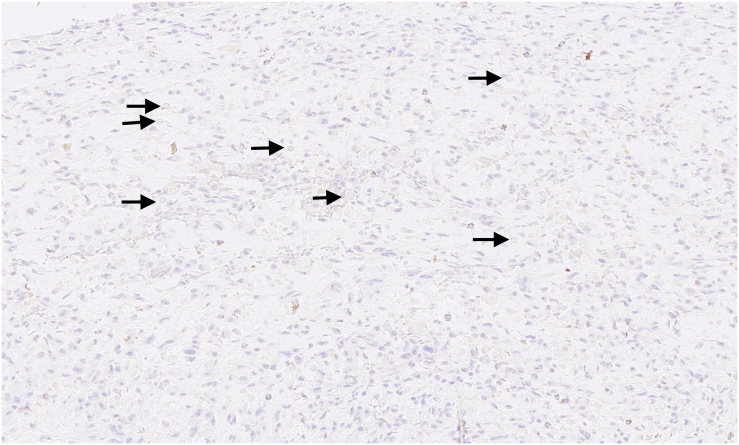
Immunohistochemical of the lesion and the inclusion bodies (arrows). Negativity for myogenin (×20).

**Fig. 10:**
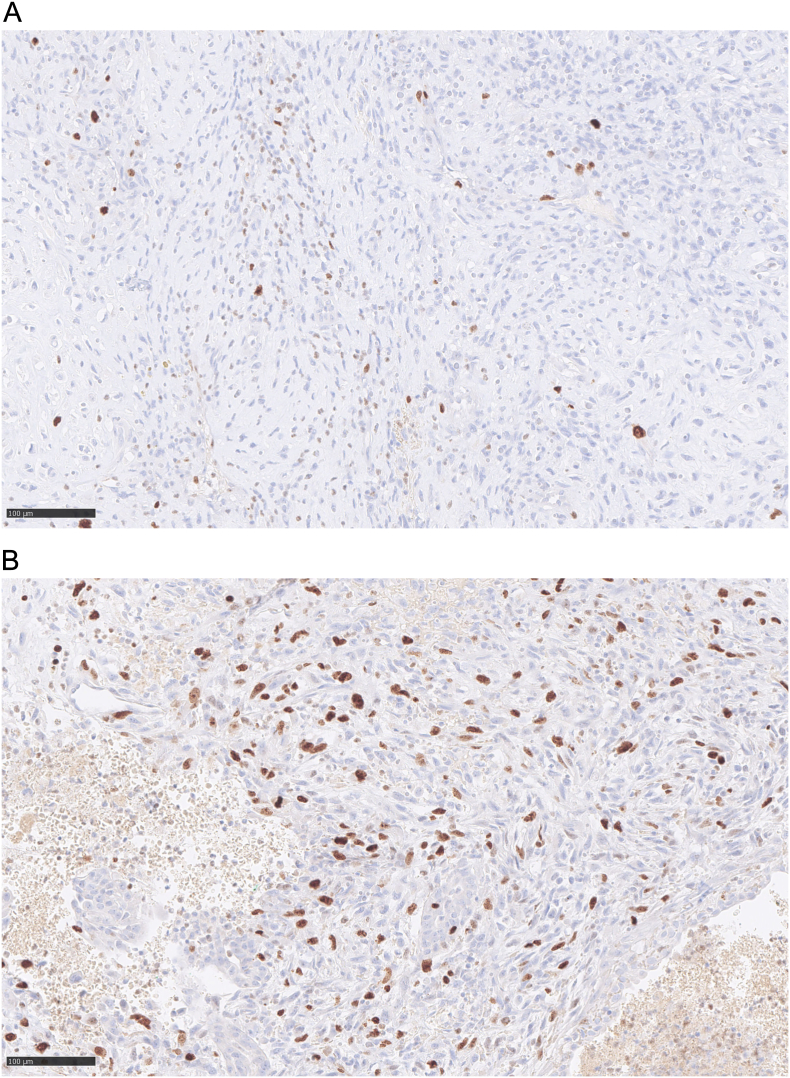
Proliferation index evaluated by Ki67 (×20). A. Stroma. B. Area with an increased stromal cellularity.

Microscopically, the tumour was well delimited. It was composed of fibroepithelial proliferation with an increased but not diffuse stromal cellularity. The stromal cells showed foci of cells with moderate to severe atypia. The atypia were characterized by mild to moderate nuclear pleomorphism. Mitotic count was 4 to 10 mitoses based on the assessment of 10 high-power fields (40×). No stromal overgrowth was present. Neither heterologous malignant element nor necrosis was observed. At a high-power view, the proliferating spindled stromal cells contained round eosinophilic intracytoplasmic inclusion bodies (Fig. 3, Fig. 4). Those inclusion bodies stained red by Masson's trichrome (Fig. 5). Immunohistochemical study demonstrated that the stromal cells were negative for p63 (clone DAK-p63, Dako) and had a “wild type” expression for p53 (cloneDO-7, Dako). The inclusion bodies showed a ringlike positivity for smooth muscle actin (Clone 1A4, Cell Marque) and vimentin (clone V9, Dako) (Fig. 6, Fig. 7). They were negative for desmin (clone D33, Dako) and myogenin (clone F5D, Dako) (Fig. 8, Fig. 9). Other immunostainings (Calponin (Dako, clone CALP), CD117 (Dako, polyclonal), CD99 (Dako, clone 12E7), EPCAM (Dako, clone Ber-EP4), MLH1 (Dako Agilent, clone ES05), PMS2 (Dako, clone EP51), MSH2 (Dako, clone FE11), MSH6 (Dako, clone EP49)) were performed. The inclusions bodies showed a ringlike positivity for calponin and they were negative for the other immunostainings (Appendix: Fig. a, Fig. b, Fig. c, Fig. d, Fig. e, Fig. f, Fig. g, Fig. h). The proliferation index evaluated by Ki67 (MIB-1, Dako) in the stroma was estimated at 5 %. However in the areas with an increased stromal cellularity the proliferation index was estimated at 10 % (Fig. 10).

The lesion was excised with a microscopic free margins. The distance between the tumour and the closest margin was evaluated at 2 mm.

The diagnosis of borderline phyllodes tumour with eosinophilic inclusion bodies was made.

Next-Generation Sequencing (NGS) targeting stromal cells with inclusions was done. The library construction was performed using two custom Ampliseq panel targeting in 58 genes (Table 1). Sequencing was performed using the Ion S5 GeneStudio instrument (ThermoFisher Scientific, Waltham, MA). Molecular tests revealed no mutations.

**Table 1:** Next-generation sequencing: library construction using 2 custom Ampliseq panels targeting 58 genes.

ABL1	AKT1	ALK	APC	ATM	AXIN1	BRAF	CDH1	CDKN2A	CHEK2
CSF1R	CTNNB1	EGFR	EIF1AX	ERBB2	ERBB4	EZH2	FBXW7	FGFR1	FGFR2
FGFR3	FLT3	GNA11	GNAQ	GNAS	HNF1A	HRAS	IDH1	IDH2	JAK2
JAK3	KDR	KIT	KRAS	MET	MLH1	MPL	NOTCH1	NPM1	NRAS
PDGFRA	PIK3CA	PPM1D	PRKAR1A	PTEN	PTPN11	RASAL1	RB1	RET	SMAD4
SMARCB1	SMO	SRC	STK11	TERT	TP53	TSHR	VHL		

## 3 Discussion and conclusion

Intracytoplasmic eosinophilic inclusion is a pathognomonic feature in infantile digital fibromatosis (IDF) that was first described by Reye in 1965 [6]. IDF is a dermal fibroblastic and myofibroblastic lesion typically found as firm nodules on digits of infants and children. Intracytoplasmic inclusion bodies in stromal cells are a classic histopathologic finding and can be highlighted with special stains like Masson's trichrome [1]. However their presence in extradigital fibromatosis and in adults is rare. Fewer than 20 cases were described. Some authors have reported intracytoplasmic inclusion bodies in cervical polyps [2], in leiomyosarcoma [3] or in phyllodes tumour of the breast [4]. In the literature, 5 cases were described in phyllodes tumour of the breast. The clinical and histological features of these cases were described in Table 2.

**Table 2:** Cases of phyllodes tumour with eosinophilic inclusion bodies in the literature.

Case	Age	Clinical presentation	Mammogram	Benign/Borderline/Malignant	Immunohistochemical stains	Electron microscopy (IB)	NGS
*Hiraoka N and al. (7)*	42	Painless, slow-growing tumour of the right breast Size: 4.9 cm	_	Benign	Stromal component: (+) actin, desmin, vimentin Inclusion bodies: (+) actin	Bundles of microfilaments	_
*Ozerdem U and al. (8)*	39	Painless, solitary, firm mass of the right breast Size: 3 cm	_	Benign	Stromal component: (+) desmin, myosin Inclusion bodies: (+) desmin, myosin	Aggregates of actin filaments	_
*Ortega E and al. (9)*	42	Quick growing, painless nodule of the right breast Size: 10 cm	Dense and well-delimited nodule, without spicula or micro-calcification	Benign	Stromal component: (+) desmin, SMA, vimentin Inclusions bodies: (+) SMA	_	_
*Harigopal M and Hosa SA. (10)*		A right breast mass Size: 3 cm	_	Benign	Inclusion bodies (−): SMA	Extremely dense actin filaments.	_
*Dey D and al. (4)*	50	Smooth lump of the left breast Size: 2 cm	Well-defined, low-density opacity	Benign	Stromal component: (+) desmin, SMA, vimentin Inclusion bodies: (+) SMA	_	_
*Our current case*	33	Mass in the upper quadrant of the right breast Size: 8 cm	Well-delimited, rare microcalcifications but not suspicious	Borderline	Stromal component: (+): desmin, SMA, vimentin Inclusion bodies: (+): SMA, vimentin & (−) desmin, myogenin	_	Panel of 58 genes: no mutation

All the 5 cases were diagnosed as benign phyllodes tumour [4,[7], [8], [9], [10]]. Our case is the first one to describe a borderline phyllodes tumour with those inclusion bodies. Ortega and al. reported a case of benign phyllodes tumour of the breast with inclusion bodies by fine-needle aspiration (FNA) [9]. Dey and al. described a case where the needle core biopsy (NCB) showed epithelial lobular units with spindled stromal cells [4]. They found multiple intracytoplasmic, homogenous eosinophilic inclusions in the spindled stromal cells. Their diagnosis was phyllodes tumour with intracytoplasmic inclusions.

The inclusion bodies in our case were positive for SMA and vimentin. In different published cases, the spindled stromal cells are positive for SMA, desmin or vimentin [4,7,8,11,12]. In contrast there is a difference between cases in positivity for SMA, with either a positivity within the inclusion or at its periphery [11,12]. The principal differential diagnosis is a rhadoid differentiation in phyllodes tumour [13]. In rhadoid differentiation there is a cohesive large epithelioid cells with irregular nuclei, prominent nucleoli and a large paranuclear intracytoplasmic eosinophilic inclusions. Most of the time there is a positive staining of myogenin on those cells. In contrast our lesion was composed of spindled stromal cells containing round eosinophilic intracytoplasmic inclusion bodies. Also the rhabdoid differentiation was invalidated by the negative staining of myogenin in our case.

Some studies performed electronic microscopy on the inclusion bodies [7,8,10]. The core of those was composed of granular material; while in the periphery, it showed a microfibrillary structure. Actin positivity and fibrillary appearance on ultrastructural examination suggest a mechanism of disturb regulation of proliferating myofibroblastic cells. We did not perform electronic microscopy but our laboratory realised molecular test of the lesion. To the best of our knowledge, molecular sequencing by NGS have not yet been conducted for lesions with inclusion bodies. We tried to analyse the area in which the inclusions were numerous. NGS (panels of 58 genes) revealed no mutations. In the literature, genetic alterations of TERT promoter and MED12 are usually observed in phyllodes tumour [14]. In borderline or malignant phyllodes tumour TP53 alteration and/or EGFR amplification can be found. In our case there were no alteration of TP53, TERT promoter and no EGFR amplification and we could not test the presence of a MED12 mutation with our panel.

The presence of inclusion bodies in breast tumour do not seem to have a prognosis impact. In the literature only one case reported a patient's follow-up. She underwent a surgery for a fibroepithelial tumour of the breast with inclusion bodies and there was no recurrence 5 years after surgery [3].

In the future, it might be interesting to perform molecular tests on lesions with eosinophilic inclusion bodies to discover potential mutations.

## Ethics statement and consent to participate

Written informed consent was obtained from the patient for publication and any accompanying images. A copy of the written consent is available for review by the Editor-in-Chief of this journal on request.

## Funding

The authors have not declared a specific grant for this research from any funding agency in the public, commercial or not-for-profit sectors.

## Declaration of competing interest

The authors declare that they have no known competing financial interests or personal relationships that could have appeared to influence the work reported in this paper.

## Figures and Tables

**Fig. a:**
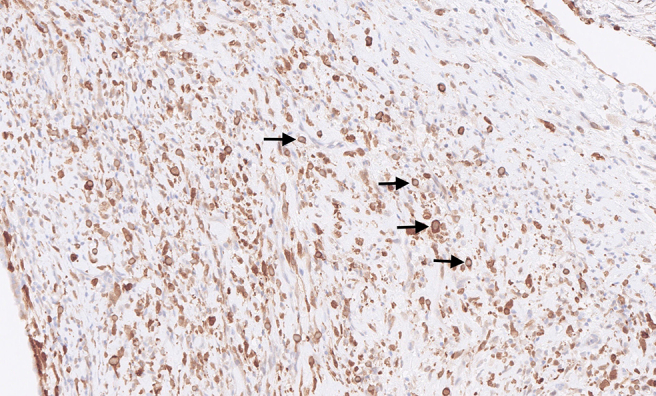
Immunohistochemical of the lesion and the inclusion bodies (arrows). Positivity for Calponin (×20).

**Fig. b:**
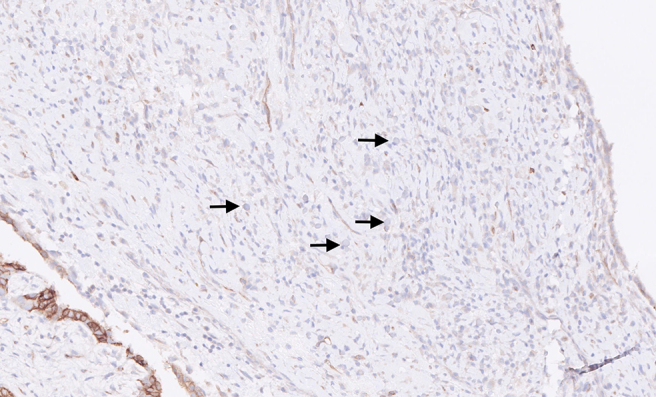
Immunohistochemical of the lesion and the inclusion bodies (arrows). Negativity for CD117 (×20).

**Fig. c:**
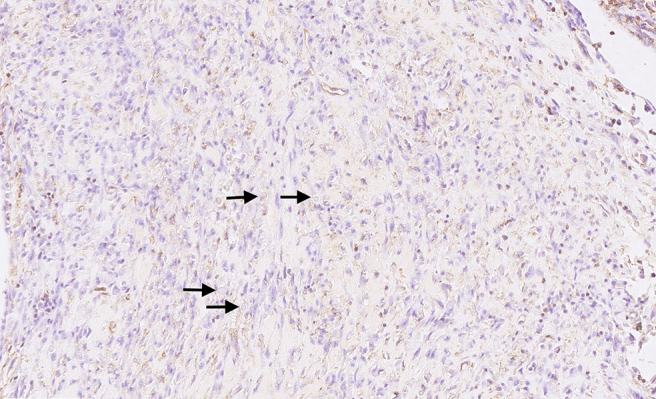
Immunohistochemical of the lesion and the inclusion bodies (arrows). Negativity for CD99 (×20).

**Fig. d:**
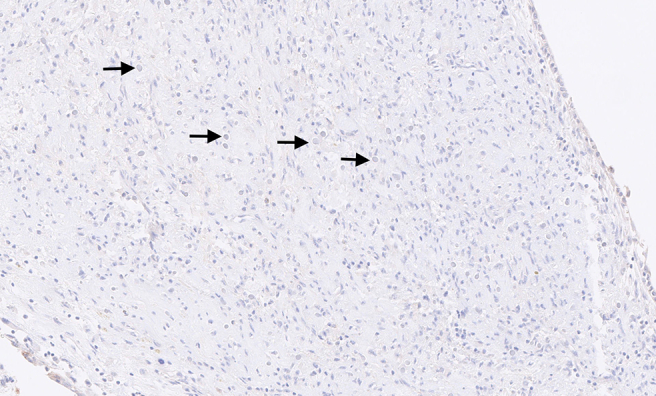
Immunohistochemical of the lesion and the inclusion bodies (arrows). Negativity for EPCAM (×20).

**Fig. e:**
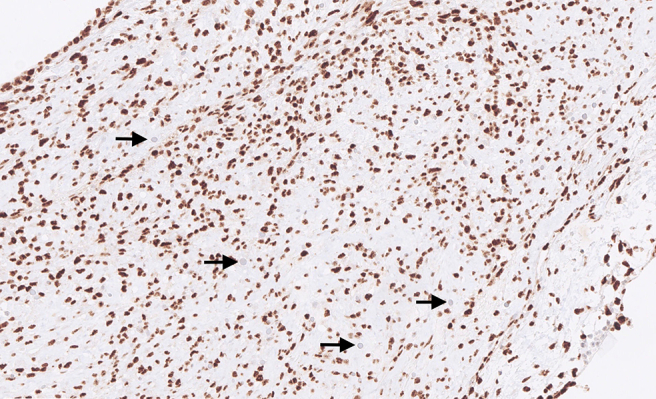
Immunohistochemical of the lesion and the inclusion bodies (arrows). Negativity for MLH1 (×20).

**Fig. f:**
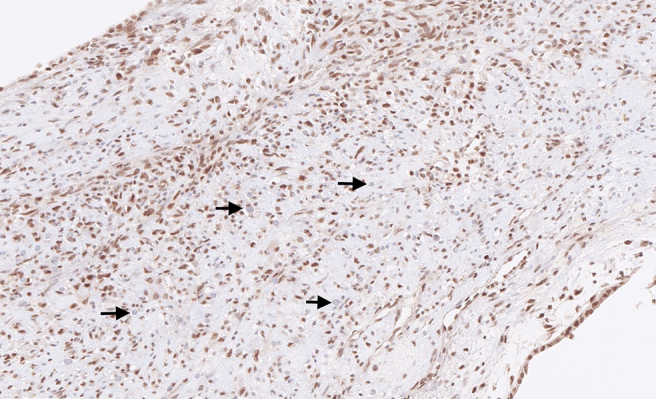
Immunohistochemical of the lesion and the inclusion bodies (arrows). Negativity for PMS2 (×20).

**Fig. g:**
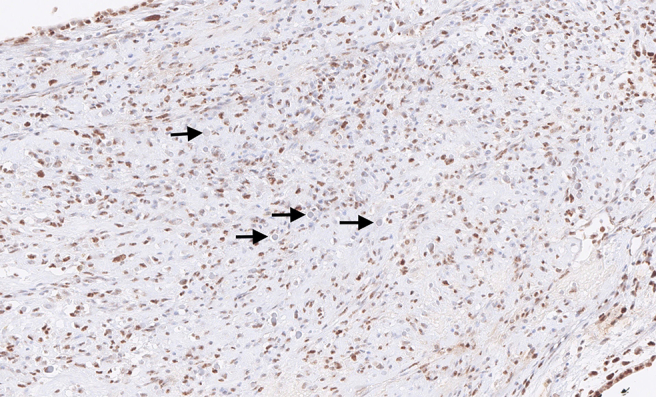
Immunohistochemical of the lesion and the inclusion bodies (arrows). Negativity for MSH2 (×20).

**Fig. h:**
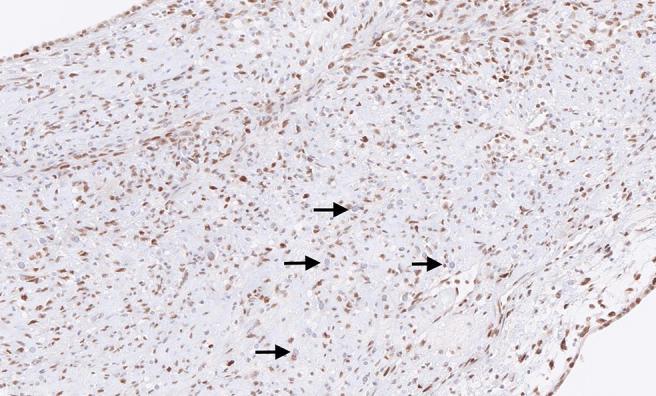
Immunohistochemical of the lesion and the inclusion bodies (arrows). Negativity for MSH6 (×20).

## Data Availability

Not applicable.
